# Evolutionary insights and expression dynamics of the CaNFYB transcription factor gene family in pepper (*Capsicum annuum*) under salinity stress

**DOI:** 10.3389/fgene.2023.1288453

**Published:** 2023-11-02

**Authors:** Diaa Abd El Moneim, Hassan Mansour, Rana M. Alshegaihi, Fatmah Ahmed Safhi, Khairiah Mubarak Alwutayd, Rahma Alshamrani, Amnah Alamri, Wessam Felembam, Amani Omar Abuzaid, Mahmoud Magdy

**Affiliations:** ^1^ Department of Plant Production (Genetic Branch), Faculty of Environmental Agricultural Sciences, Arish University, El-Arish, Egypt; ^2^ Department of Biological Sciences, Faculty of Science & Arts, King Abdulaziz University, Rabigh, Saudi Arabia; ^3^ Department of Botany and Microbiology, Faculty of Science, Suez Canal University, Ismailia, Egypt; ^4^ Department of Biology, College of Science, University of Jeddah, Jeddah, Saudi Arabia; ^5^ Department of Biology, College of Science, Princess Nourah bint Abdulrahman University, Riyadh, Saudi Arabia; ^6^ Department of Biological Sciences, King Abdulaziz University, Jeddah, Saudi Arabia; ^7^ Immunology Unit, King Fahad Medical Research Centre, King Abdulaziz University, Jeddah, Saudi Arabia; ^8^ Department of Genetics, Faculty of Agriculture, Ain Shams University, Cairo, Egypt

**Keywords:** CaNFYB transcription factor, gene structure, phylogenetic analysis, expression analysis, salinity stress, *Capsicum annum*

## Abstract

**Introduction:** The *Capsicum annuum* nuclear factor Y subunit B (CaNFYB) gene family plays a significant role in diverse biological processes, including plant responses to abiotic stressors such as salinity.

**Methods:** In this study, we provide a comprehensive analysis of the CaNFYB gene family in pepper, encompassing their identification, structural details, evolutionary relationships, regulatory elements in promoter regions, and expression profiles under salinity stress.

**Results and discussion:** A total of 19 CaNFYB genes were identified and subsequently characterized based on their secondary protein structures, revealing conserved domains essential for their functionality. Chromosomal distribution showed a non-random localization of these genes, suggesting potential clusters or hotspots for NFYB genes on specific chromosomes. The evolutionary analysis focused on pepper and comparison with other plant species indicated a complex tapestry of relationships with distinct evolutionary events, including gene duplication. Moreover, promoter cis-element analysis highlighted potential regulatory intricacies, with notable occurrences of light-responsive and stress-responsive binding sites. In response to salinity stress, several CaNFYB genes demonstrated significant temporal expression variations, particularly in the roots, elucidating their role in stress adaptation. Particularly *CaNFYB01*, *CaNFYB18*, and *CaNFYB19*, play a pivotal role in early salinity stress response, potentially through specific regulatory mechanisms elucidated by their cis-elements. Their evolutionary clustering with other Solanaceae family members suggests conserved ancestral functions vital for the family’s survival under stress. This study provides foundational knowledge on the CaNFYB gene family in *C. annuum*, paving the way for further research to understand their functional implications in pepper plants and relative species and their potential utilization in breeding programs to enhance salinity tolerance.

## 1 Introduction

Salinity stress exerts a significant global impact on pepper (*Capsicum* spp.) cultivation, with regions facing elevated soil salinity due to factors such as coastal proximity, suboptimal irrigation practices, or saline water usage experiencing the most significant challenges. The consequences of salinity stress on pepper crops encompass both yield losses and quality degradation. Research has indicated that the severity of salinity can lead to substantial yield reductions, ranging from 20% to over 50%, contingent on the specific pepper variety and local conditions (Semiz et al., 2014; Parmar et al., 2017). Furthermore, the quality of the harvested fruits is compromised under salinity stress, with noticeable alterations in flavour, texture, colour, and nutritional content (Savvas and Gruda, 2018). This issue is particularly pertinent in arid or semi-arid regions, including the Mediterranean, the Middle East, certain parts of Asia, and coastal areas, where pepper cultivation faces increased susceptibility to salinity-related challenges (Munns and Tester, 2008). The economic consequences of salinity-induced yield losses and reduced fruit quality are significant, impacting the livelihoods of farmers and potentially leading to elevated market prices due to diminished supply (Chinnusamy et al., 2005). In response, the agricultural community has initiated research to develop salt-tolerant pepper varieties, improve cultivation techniques, and adopt precision irrigation strategies to mitigate these challenges (Brindha et al., 2021). Therefore, it is essential to understand how pepper responds and withstand such challenges (Chen et al., 2015).

Previous studies examining the effect of salinity on pepper plants, both at the molecular and production levels, have highlighted the multifaceted impact of this stress on the crop. The effect of salinity stress on pepper production has been investigated by previous research (Leyva-González et al., 2012). This study elucidated the impact of varying levels of salinity on pepper crop yield, quality, and overall plant health. It demonstrated that salinity stress led to reduced fruit yield, altered fruit size, and compromised nutrient uptake, ultimately affecting the economic viability of pepper cultivation. At the molecular level, Jia et al. (2016) have provided valuable insights into the changes in gene expression patterns in response to salinity stress in pepper plants. They identified key genes involved in ion transport, osmotic regulation, and stress signaling pathways, shedding light on the intricate network of molecular responses triggered by salt stress. Such findings emphasize the need to explore the role of transcription factors, like the NFYB gene family, which likely serve as central regulators orchestrating these complex responses (Irikova et al., 2012).

The NFYB (Nuclear Factor Y, subunit B) gene family belongs to a group of transcription factors known as the Nuclear Factor Y (NF-Y) complex. The NF-Y complex is a heterotrimeric transcription factor composed of three subunits: NFYA, NFYB, and NFYC. NFYB (also referred to as NF-YB/NF-YC-like) is one of the subunits that form this complex, and it plays a crucial role in the regulation of gene expression in eukaryotes (Laloum et al., 2013). The NFYB subunit, in association with the other subunits of the NF-Y complex, binds to specific DNA motifs known as the CCAAT boxes, commonly found in the promoters of many genes. This binding facilitates the initiation of transcription, making the NF-Y complex a central player in the regulation of various cellular processes, including growth, development, stress responses, and cell cycle control (Rushton et al., 2008). Numerous past studies have shed light on the importance of NFYB genes in plants. A study by Leyva-González et al. (2012) in *Arabidopsis thaliana* revealed that NFYB genes play crucial roles in modulating ABA-mediated stress responses, including salinity stress. This study demonstrated the involvement of NFYB genes in regulating stress-responsive gene expression and their impact on stress-related phenotype. Additionally, research by Dai et al. (2021) on *Eucalyptus grandis* highlighted the significance of NFYB transcription factors in salt and temperature stress tolerance.

The presence of reference pepper genomes (Kim et al., 2014; Qin et al., 2014; Magdy et al., 2019; Magdy and Ouyang, 2020) provide a framework for identifying and functionalizing a gene family *in silico*. Thus, enable the integration of molecular insights with production-level which is crucial for developing effective strategies to mitigate the adverse effects of salinity stress. Given the relevance of the NFYB gene family in stress responses, including salinity stress, and the potential for crop improvement, our study investigates the NFYB gene family in pepper (*Capsicum* spp.) under salinity stress conditions. By elucidating the molecular mechanisms through genome-wide identification analysis and validating the expression patterns using qPCR, we aim to understand how the NFYB gene family contributes to salt tolerance in pepper can potentially address these production-level challenges by providing molecular targets for breeding or genetic engineering efforts aimed at improving salinity tolerance.

## 2 Material and methods

### 2.1 Identification of members of the CaNFYB gene family in pepper

Structural domains of NFYB genes from *A. thaliana* were sourced from the TAIR.org database. These sequences served as probes in homology searches via BLASTp to mitigate potential omissions of sequences with limited similarity to the probes, as suggested by Wang et al. (2022). This was applied to the most recent pepper reference genomics on the NCBI database (UCD10Xv1.1) and was further compared against previous versions of pepper genomes and the plant transcription factor database at plantTFdb.org. To validate the functional attributes and accuracy of the obtained sequences, the presence of the histone-like transcription factor CBF/NFY domain (pfamID: PF00808, InterPro ID: IPR003958) was inspected through InterProScan (accessible at: https:// www.ebi.ac.uk/interpro/). Physiochemical characteristics of the CaNFYB genes were deduced using the Expasy online platform (accessible at: http://web.expasy.org/). Subcellular localization analysis for the CaNFYB gene family in pepper was conducted with the online tool Cell-Ploc 2.0 (accessible at: http://www.csbio.sjtu.edu.cn/bioinf/Cell-PLoc-2/ affirming its nuclear localization. Conclusively, SPOMA online software was deployed to forecast the secondary structures of CaNFYB proteins (Sapay et al., 2006), with validation sought via the Protein Data Bank (PDB) search.

### 2.2 Chromosomal location, gene structure, cis-element analysis in promoter regions

The positional distribution of CaNFYB genes on the pepper chromosomes was determined using the MG2C V2 tool (Chao et al., 2021). The structural architecture of these genes was further elucidated through the visualization features and annotation tools in Geneious Prime software (Kearse et al., 2012). Additionally, genomic sequences extending 1.5 kilobases upstream of the initiation codon for each gene were extracted from the reference genome and subsequently analyzed for potential cis-regulatory elements using the PlantCARE database (accessible at: http://bioinformatics.psb.ugent.be/webtools/plantcare/html/).

### 2.3 Evolutionary analysis of the CaNFYB gene family

To elucidate the evolutionary trajectories of the NFYB transcription factors, members of the NFYB gene family from diverse species were procured through the employment of the local BLAST computational methodology. The protein sequences of the CaNFYB gene family were subsequently aligned with the NFYB genes derived from *Physcomitrium patens* (7 gene copies, reference: Phypa-V3 from Ensemble Plants), *A. thaliana* (12 gene copies, sourced from TAIR.org), as well as two congeneric taxa from the Solanaceae family, specifically *Solanum lycopersicum* (18 gene copies, reference: SL4.0, accessible at https://solgenomics.net/) and *Solanum tuberosum* (20 gene copies, reference: SolTub_3, accessible at https://solgenomics.net/).

Alignment of the sequences was executed utilizing the MAFFT aligner algorithm as delineated by Katoh and Standley (2013), incorporated within the Geneious Prime. This was succeeded by the construction of phylogenetic trees through the application of maximum likelihood methodologies (ML). The computation of the ML tree was facilitated through the employment of FastTree V2 integrated within the Geneious Prime framework, adhering to the Jones-Taylor-Thorton (JTT) evolutionary model. For designating the root of the tree, a preliminary ancestral sequence inference was conducted. This was subsequently complemented by an analysis of gene duplication events using MEGA 11 software (Tamura et al., 2021).

### 2.4 Plant materials and salinity stress treatment

Seeds from the pepper cultivar Gedeon F1 (*C. annuum* L., syngenta. com.eg) underwent sterilization using 1% sodium hypochlorite for 30 min, and post-sterilization, were rinsed with sterile water before sowing in perlite beds at 28°C, adopting the method detailed by (Qin et al., 2014). Following germination, seeds were transplanted into pots (containing a mixture of peat moss, perlite, and vermiculite in equal proportions) and cultivated under a regimen of 16 h of light at 25*°*C and 8 h of darkness at 18*°*C, maintaining a relative humidity of 60%. Seedlings were considered mature upon the development of their sixth leaf, and subsequent irrigation was conducted using a half-strength Hoagland solution, adjusted to a pH of 5.6. Leaf and root samples were procured from these seedlings, with each sampling event being triplicated for control measures. For salinity stress assessments on five-week-old plants, irrigation was carried out using a 200 mM NaCl solution (Wang et al., 2020). Sampling intervals post-treatment were set at 12 and 24 h, where each sample, comprising three to four leaves, was collected in triplicate. To preserve sample integrity, immediate freezing with liquid nitrogen was employed, with long-term storage facilitated at −80*°*C until RNA extraction commenced. A two-way ANOVA complemented by a Tukey multiple comparison *post hoc* test was applied to examine the gene expression changes significance using GraphPrism V9.

### 2.5 RNA expression and qPCR analysis

Total RNA was extracted from pepper tissues, inclusive of leaves and roots, subject to varying salt stress treatments, utilizing the EasyPure^®^ Plant RNA Kit (TransGen Biotech, Beijing, China) as instructed by the manufacturer. The resultant RNA was evaluated both qualitatively and quantitatively via 2% agarose gel electrophoresis and the Quantus^TM^ Fluorometer (Promega, United States). The reverse transcription of RNA to cDNA was facilitated by the SuperScript III reverse transcriptase (Invitrogen, Carlsbad, CA, United States), yielding concentrations adjusted to 100 ng *µ*L-1. PCR amplifications spanned sequences ranging from 95 to 245 bases, deliberately circumventing the conserved region, as detailed in Supplementary Table S1). For quantitative real-time RT-PCR (qRT-PCR) procedures, the TransStart^®^ Green qPCR SuperMix (TransGen Biotech, Beijing, China) was deployed. Amplification conditions were set to an initial 95*°*C for 5 min, followed by 40 cycles of sequential temperatures: 95*°*C for 15 s, 55*°*C for 20 s, and 72*°*C for 30 s. Subsequent melting curve analysis, which entailed a temperature ramp from 55*°*C to 95*°*C, and gel electrophoresis confirmed the singular amplicon nature of the products. Fold differences in each assay were determined by the ∆∆Ct method, with normalization anchored to GAPDH. All tests were conducted in duplicate for each sample.

## 3 Results

### 3.1 Identification of the CaNFYB family genes members in pepper

A comprehensive analysis revealed the identification and annotation of a total of 19 gene copies designated as CaNFYB genes from the UCD10Xv1.1 reference genome. This stands in notable contrast to the fewer gene copies with different genome position and transcription direction reported in earlier published versions: specifically, 15 copies in the PlantTF database, 16 copies in the Zunla V2 NCBI database, and 18 copies in the ASM51225v2 Ensembl Plant database (Supplementary Table S2). The observed variation in the number of gene copies across these versions underscores the evolving nature of gene annotations and the significance of utilizing the most recent reference genome for accurate gene identification, thus the final 19 CaNFYB gene copies were used for further analysis.

A summary of key biochemical properties and domain information for CaNFYB genes in pepper is shown in Table 1. Among the studied NFYB genes, the gene with the highest molecular weight (Mw) is *CaNFYB16* with a value of 27.32 kDa. On the other hand, the gene with the lowest Mw is *CaNFYB09*, which has a molecular weight of 16.70 kDa. In terms of isoelectric point (pI), *CaNFYB06* exhibits the highest value at 6.62, signifying a more basic characteristic. Conversely, *CaNFYB01* and *CaNFYB07* share the lowest pI values of 4.49, indicating a more acidic nature. Regarding the charge at physiological pH (pH7), the most positively charged gene is *CaNFYB04* with a value of 0.89, in contrast, *CaNFYB07* demonstrates the most negative charge with a value of −12.13. The gene with the highest extinction coefficient is *CaNFYB13*, with a value of 23,045, indicating strong absorbance characteristics. While *CaNFYB07* has the lowest extinction coefficient at 4,720, suggesting relatively weaker absorbance. The annotated CBF/NF-Y domain length varied among copies. The longest domain is observed in *CaNFYB10*, spanning 134 amino acids, while the shortest domain is present in *CaNFYB14*, covering only 48 amino acids. This disparity in domain length suggests varying functional complexities among the NFYB gene isoforms. All copies were confirmed to localize in the nucleus.

**TABLE 1:** Physicochemical Properties and Domain Characteristics of *CaNFYB* Proteins in *C. annuum*.

CaNFYB ID	nt length	AA length	Mw (kDa)	Isoelectric point	Charge at pH 7	Extinction coefficient	Domain start	Domain end	Domain length
CaNFYB01	444	147	16.65	4.49	−9.20	10,220	9	72	64
CaNFYB02	606	201	20.65	5.95	−2.71	17,545	26	90	65
CaNFYB03	543	180	19.52	6.51	−0.81	11,585	38	102	65
CaNFYB04	564	187	21.01	7.77	0.89	20,065	30	94	65
CaNFYB05	495	164	17.72	5.00	−3.11	9,065	31	95	65
CaNFYB06	657	218	24.45	6.62	−0.71	18,575	57	121	65
CaNFYB07	477	158	17.60	4.36	−12.13	4,720	14	79	66
CaNFYB08	480	159	17.95	4.37	−12.13	10,220	14	78	65
CaNFYB09	444	147	16.70	5.23	−4.94	11,710	25	89	65
CaNFYB10	717	238	26.63	5.71	−8.95	16,180	70	134	65
CaNFYB11	510	169	18.37	4.68	−6.01	14,565	35	99	65
CaNFYB12	456	151	17.30	5.02	−5.65	17,420	2	55	54
CaNFYB13	585	194	22.01	5.73	−6.22	23,045	35	98	64
CaNFYB14	456	151	17.36	6.07	−2.55	17,420	8	55	48
CaNFYB15	561	186	20.53	5.26	−4.80	14,565	29	93	65
CaNFYB16	735	244	27.32	5.06	−9.47	21,555	78	142	65
CaNFYB17	456	151	17.37	5.90	−5.09	22,920	1	55	55
CaNFYB18	534	177	20.27	6.58	−0.915	13,200	37	101	65
CaNFYB19	534	177	20.27	6.58	−0.915	13,200	37	101	65

The table enumerates the CaNFYB gene variants, describing their length (nt and amino acid), molecular weight, isoelectric point, net charge at pH 7, and the extinction coefficient. Additionally, the domain specifications for each variant, including the start, end, and length of the domain, are provided.

### 3.2 Secondary structures analysis of CaNFYB proteins

**FIGURE 1:**
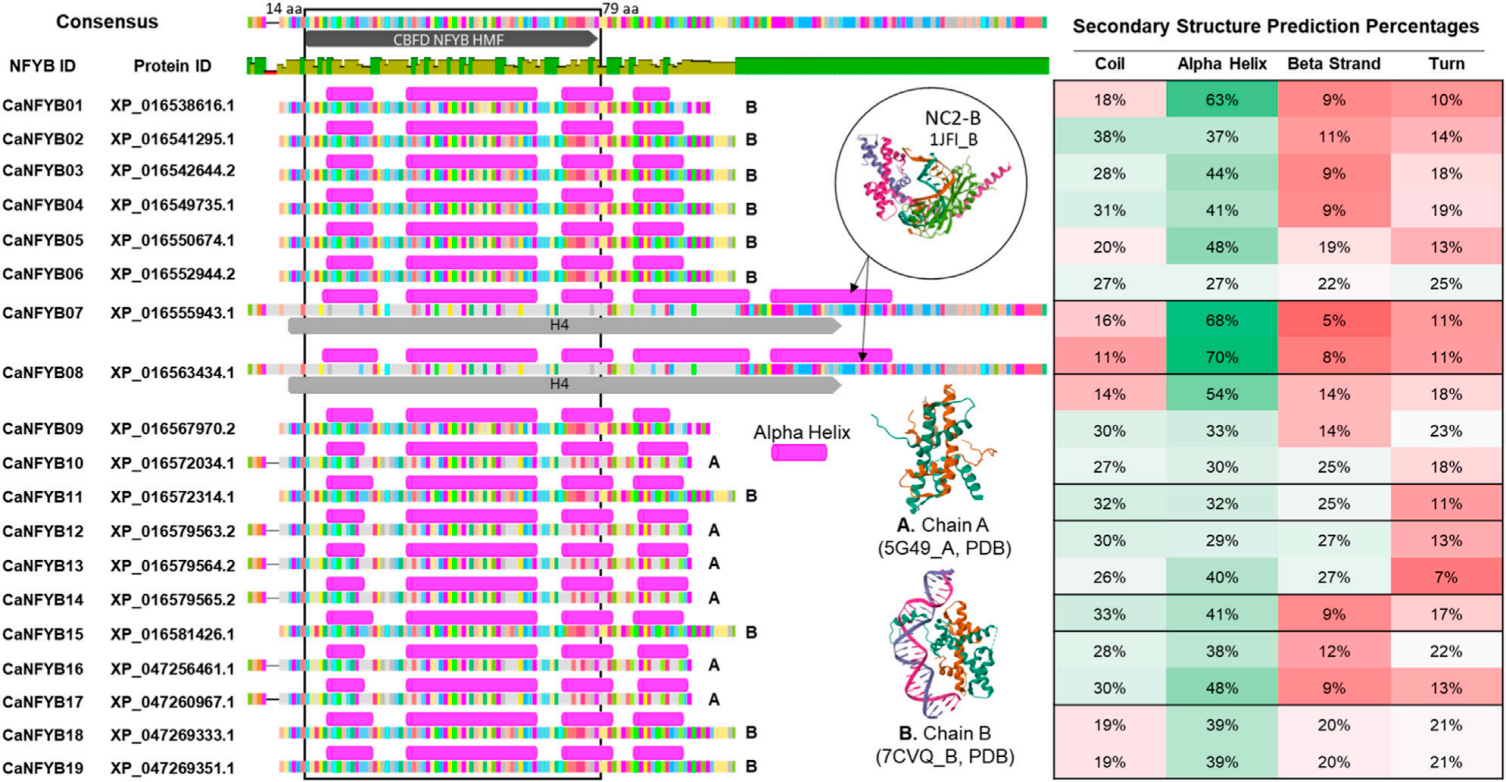
Comparative Analysis of Secondary Structures in CaNFYB Gene Variants and Reference Proteins from PDB. Secondary structural distributions of 19 CaNFYB genes grouped into A, B, and C. The predictions are correlated with structures in the Protein Data Bank: Arabidopsis histone-fold dimer L1L NF-YC3 (PDB ID: 5G49l, denoted as ‘A’), the Arabidopsis CO CCT domain-NF-YB2/YC3-FT CORE1 DNA complex (PDB ID: 7CVQ, denoted as ‘B’), and the NC2-TBP-DNA Ternary Complex, specifically NC2-B (PDB ID: 1JFI, also labeled as ‘B’). The structural compositions are shown for each group, representing the distribution of coils, alpha helices, beta strands, and turns.

The secondary structural predictions of the 19 CaNFYB genes, which possess the NFYB domain, were correlated with existing structures in the Protein Data Bank (PDB). Specifically, these genes were compared to the crystallographic structures of the Arabidopsis histone-fold dimer L1L NF-YC3 (PDB ID: 5G49l, denoted as ‘A’ in Figure 1), the Arabidopsis CO CCT domain complexed with NF-YB2/YC3 and FT CORE1 DNA (PDB ID: 7CVQ, denoted as ‘B’ in Figure 1), and the NC2-TBP-DNA Ternary Complex (specifically, NC2-B, with PDB ID: 1JFI, also labeled as ‘B’ in Figure 1).

The secondary structure analysis of the CaNFYB genes indicates a predominant presence of coils and alpha helices, with a more reserved appearance of beta strands. For Group A, which includes genes *CaNFYB09*-*10*, *CaNFYB12*-*14*, and *CaNFYB16*-*17*, the structural compositions are as follows: coils range from 14% to 32%, alpha helices span from 29% to 54%, beta strands vary between 9% and 27%, and turns are between 7% and 23%. In Group B, encompassing genes *CaNFYB01*-*06*, *CaNFYB11*, *CaNFYB15*, and *CaNFYB18*-*19*, the distributions differ: coils are found in the range of 18%–38%, alpha helices vary from 27% to 63%, beta strands range from 9% to 25%, and turns span from 10% to 25%. Finally, Group C, which includes the genes *CaNFYB07*-*08*, showcases the most pronounced differences with coils between 11% and 16%, a remarkable increase in alpha helices ranging from 68% to 70%, beta strands from 5% to 8%, and turns both at 11%.

Group C is distinguished by its high percentage of alpha helices, while Groups A and B exhibit more modest levels. In contrast, coil content is most pronounced in Group A, followed by Groups B and then C. As for beta strand content, it is most abundant in Group A, tapering off in Groups B and C. While turn percentages display slight variations, they remain generally consistent across all groups. The differences in secondary structural distributions among these groups may be attributed to multiple factors including gene length, amino acid sequence composition, and the specific molecular environment in which the protein is situated.

### 3.3 Chromosomal location and structure of CaNFYB genes

**FIGURE 2:**
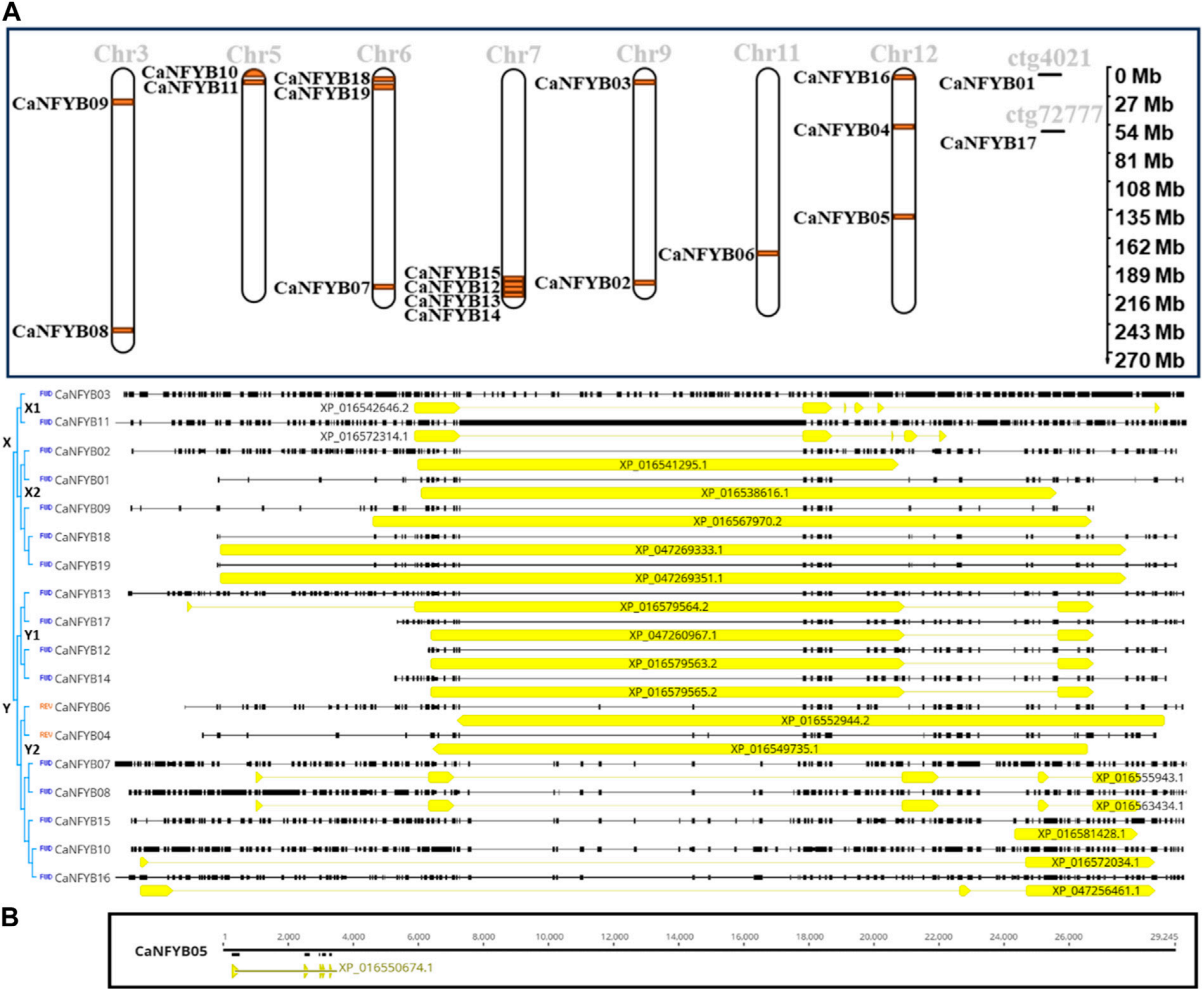
Chromosomal Distribution, Gene Length, and Phylogenetics of CaNFYB Genes. **(A)** Chromosomal distribution and transcriptional orientations of CaNFYB genes across multiple chromosomes and contigs. The data highlight distinct chromosomal loci for the genes, with several genes clustered together on the same chromosome. [**(B)**: Top] Proportion of coding DNA sequence (CDS) relative to the total annotated gene length (GL) of the CaNFYB genes and demonstrates the number of introns and exons for each gene, indicating different structural complexities. [**(B)**: Bottom] The CaNFYB05 gene, noted for its distinctness, was used as a root for tree construction but was later excluded for visual clarity.

The CaNFYB genes are distributed across an array of chromosomes, showcasing distinct chromosomal loci and transcriptional orientations. Chromosome 3 features *CaNFYB08* at about 250.7 Mb and *CaNFYB09* at roughly 32.2 Mb. Both genes are transcribed in the forward direction. On chromosome 5, *CaNFYB10* is sited at close to 2.0 Mb and is reverse-transcribed, while *CaNFYB11* is at around 10.6 Mb, transcribing in the forward direction. Chromosome 6 encompasses *CaNFYB07* at a position nearing 208.8 Mb, with *CaNFYB18* and *CaNFYB19* situated at nearly 0.8 Mb (reverse direction) and 1.2 Mb (forward direction), respectively. A cluster of genes resides on chromosome 7: *CaNFYB12*, *CaNFYB13*, *CaNFYB14*, and *CaNFYB15*, all at proximate positions around 225.1 Mb and all transcribed in the reverse direction. Chromosome 9 hosts *CaNFYB02* and *CaNFYB03* at estimated positions of 203.6 Mb and 11.5 Mb, respectively, with both oriented in the forward direction. On chromosome 11, *CaNFYB06* lies at approximately 174.5 Mb and is reverse-transcribed. Chromosome 12 is home to three genes: *CaNFYB04* at nearly 56.0 Mb (reverse direction), CaNFYB05 at about 140.2 Mb (forward direction), and *CaNFYB16* positioned at roughly 1.1 Mb, which transcribes in the forward direction. Beyond standard chromosomes, certain genes are uniquely mapped to contigs. *CaNFYB01* is located on contig ctg4021 at the 31,833 locus in the reverse direction, while *CaNFYB17* is mapped to contig ctg72777 at position 1,493, also oriented in the reverse direction (Figure 2).

A simple assessment was conducted on the proportion of coding DNA sequence (CDS) relative to the total annotated gene length (GL) for the CaNFYB genes. *CaNFYB09* and *CaNFYB18* along with CaNFYB19 demonstrated the highest CDS proportions, with each gene comprising above 75% of its total length as coding sequence, precisely 75.51%, 83.44%, and 83.44%, respectively. Meanwhile, genes such as *CaNFYB01* and *CaNFYB04* showed CDS constituting approximately 63.07% and 66.20% of their total lengths respectively. Other genes like *CaNFYB02*, *CaNFYB03*, and *CaNFYB07* had notably lower proportions, with the CDS making up 23.91%, 8.78%, and 12.99% of their respective total gene lengths. On the opposite spectrum, *CaNFYB05* displayed the lowest CDS proportion, with only about 1.69% of its total gene length being attributed to the coding sequence. The remaining genes exhibited a diverse range of CDS proportions relative to their total lengths, with number of introns ranged from 0 to 4 (exons ranged from 1 to 5), indicating varying structural complexities and possible regulatory elements interspersed within these genes (Figure 2). Upon evaluating the coding DNA sequence (CDS) of the CaNFYB genes, these genes were delineated into two primary clusters, designated as groups X and Y. This categorization was influenced by the notable sequence homology observed within these groups. Interestingly, genes within the same cluster shared analogous gene structures, a phenomenon that appeared to be independent of their chromosomal positions. The *CaNFYB05* gene, with a gene length of 29,352 bp and a coding proportion of merely 1.69%, stood out as the most distinct variant. It was judiciously chosen as the root for phylogenetic tree construction, but later omitted from the visual representation to enhance clarity of Figure 2. Within Group X, a bifurcation into two subclusters emerged. The first comprised of *CaNFYB03* and *CaNFYB11*, whereas the second encompassed genes *CaNFYB01*-*02*, *CaNFYB09*, and *CaNFYB18*-*19*. Analogously, in Group Y, two distinct subclusters were identified. The first subset integrated genes *CaNFYB12*-*14* and *CaNFYB17*. The second, however, exhibited a more intricate layout, with one of its minor clades presenting a reverse complement sequence alignment of the *CaNFYB04* and *CaNFYB06* genes. The other minor clade within this subcluster assembled genes *CaNFYB07*-*08*, *CaNFYB10*, and *CaNFYB15*-*16* (Figure 2).

### 3.4 Potential cis-element analysis in promoter regions of CaNFYB genes

**FIGURE 3:**
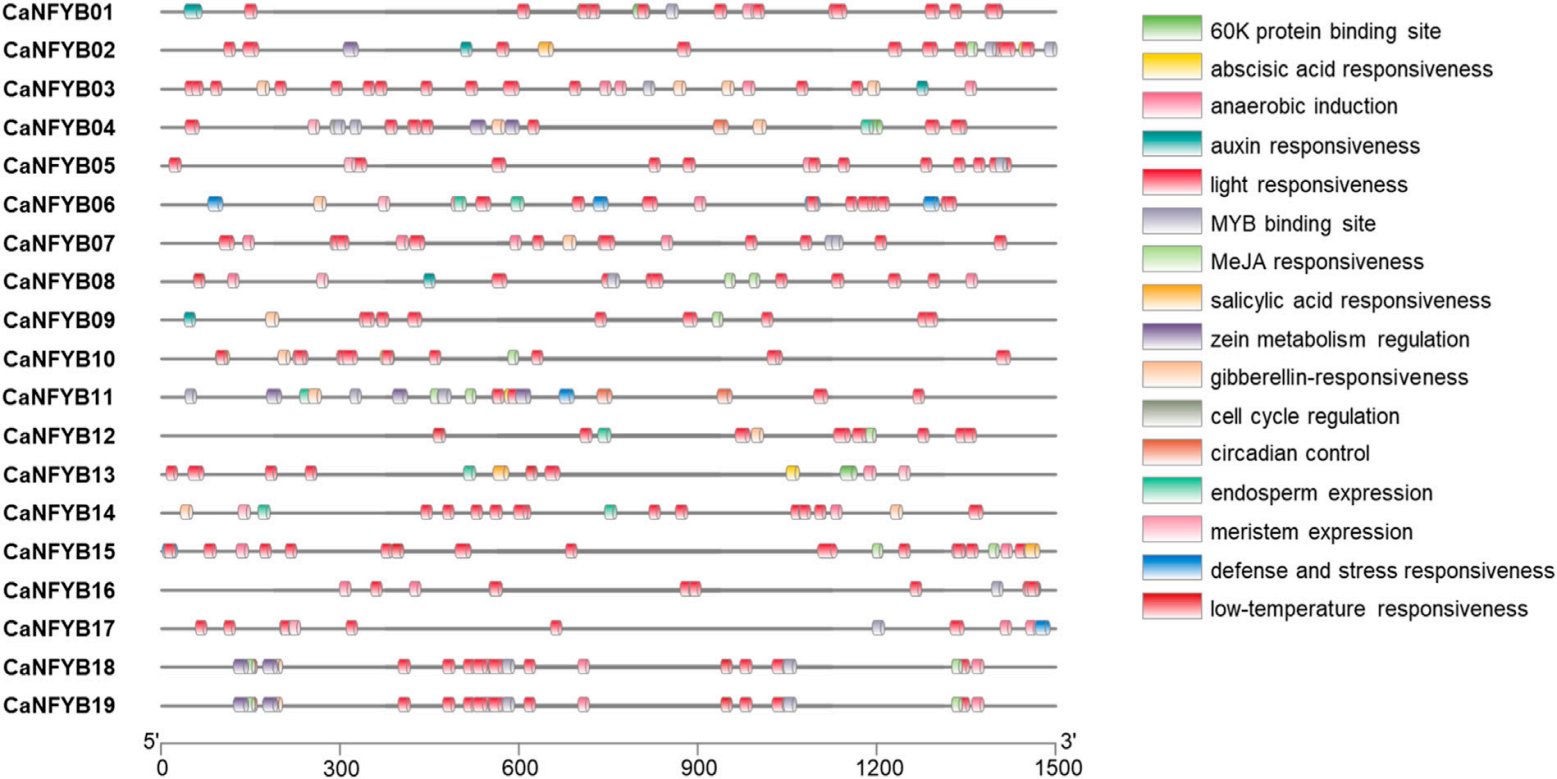
Distribution of transcription factor (TF) binding sites in the 1,500 bp upstream sequences from the translation start sites of CaNFYB genes. Multiple categories of responsiveness are showcased, including light, abscisic acid, MeJA, and anaerobic induction, among others. Salinity-related TF binding sites can be inferred from categories like abscisic acid responsiveness, defense and stress responsiveness and salicylic acid responsiveness, which have been linked with salinity response in plants.

To further characterize the potential regulatory mechanisms of CaNFYB, 1,500 bp upstream sequences from the translation start sites were analyzed. The transcription factor (TF) binding site analysis of CaNFYB genes revealed specific distribution patterns related to various physiological and developmental processes. Among the genes, light responsiveness binding sites were the most prevalent, with a cumulative count of 205 across all the genes. The genes *CaNFYB18* and *CaNFYB19* each had 12 sites associated with this category. Meanwhile, anaerobic induction had a total of 28 occurrences, with *CaNFYB03* and *CaNFYB07* both displaying 4 sites. *CaNFYB18* and *CaNFYB19* emerged as the genes with the most diversified TF binding sites, each registering a total of 30. On the other hand, *CaNFYB05* and *CaNFYB09* exhibited lower binding site diversity, registering totals of 15 and 14, respectively. A unique observation was made for *CaNFYB11*, which was the sole gene associated with a binding site for palisade mesophyll cells differentiation. The cumulative data paints a detailed landscape of the potential regulatory intricacies governing the CaNFYB genes (Figure 3).

The transcription factor binding sites associated with the CaNFYB genes in pepper reveal insights into potential salinity responses. Salinity-related TF binding sites can be inferred from categories like “abscisic acid responsiveness”, “defense and stress responsiveness,” and “salicylic acid responsiveness”, which have been linked with salinity response in plants. For instance, *CaNFYB01* and *CaNFYB02* show prominent abscisic acid-responsive binding sites, with *CaNFYB02* also having a salicylic acid-responsive site. *CaNFYB08* and *CaNFYB10* exhibit notable abscisic acid responsiveness, with *CaNFYB10* also responding to MeJA, another stress-related molecule. *CaNFYB11* stands out with multiple sites for both abscisic acid and MeJA responsiveness, while *CaNFYB12* and *CaNFYB13* also indicate a salinity response via abscisic acid-responsive sites. *CaNFYB15* and *CaNFYB16* emphasize abscisic acid-related sites, and *CaNFYB18* and *CaNFYB19* have an array of binding sites for abscisic acid, MeJA, and defense and stress responsiveness. Collectively, these genes possess 45 abscisic acid-responsive sites, 7 defense and stress-responsive sites, and 3 salicylic acid-responsive sites, highlighting the potential role of CaNFYB genes in salinity responses in pepper (Figure 3).

### 3.5 Evolutionary analysis of CaNFYB genes

**FIGURE 4:**
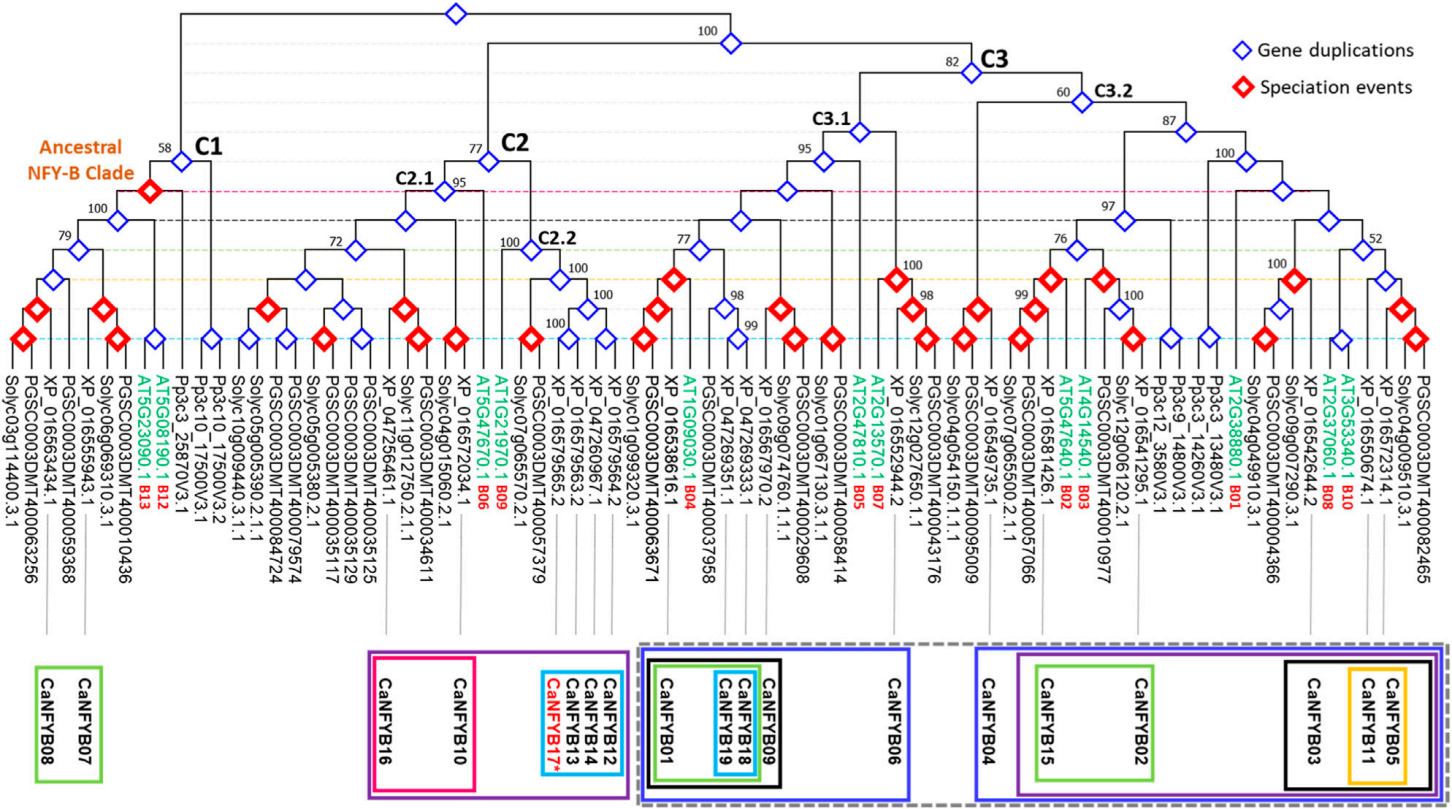
Phylogenetic tree depicting evolutionary relationships of NFYB genes from Arabidopsis (AT), potato (PGSC), tomato (Solyc), moss (Pp), and pepper (XP). The tree is rooted using the moss gene Pp3c10_17500V3.2. The tree unravels evolutionary nuances, gene duplications, and potential shared ancestral functions in the NFYB gene family across the studied plants.

The NFYB transcription factor family phylogenetic tree represents a complex tapestry of evolutionary relationships among genes from *A. thaliana* (AT), potato (*S. tuberosum*, PGSC), tomato (*S. lycopersicum*, Soly), the moss *Physcomitrella patens* (Pp), and pepper (XP), with a particular focus on pepper. The tree was rooted by the clade containing the most ancestral gene copy (Pp3c10_17500V3.2; *P. patens*) causing the tree to bifurcate into two additional predominant clades. This split indicates a major evolutionary event or divergence point, where the ancestral gene might have duplicated or otherwise diverged (Figure 4).

In the ancestral clade (C1), described according to Arabidopsis genes AT5G08190.1 and AT5G23090.1 as B12/B13 subunits clade, *CaNFYB*07 and *CaNFYB08* genes cluster closely with both tomato and potato sequences independently while the Arabidopsis genes, though distinct, weave into this cluster, suggesting parallel evolutionary paths or shared ancestral functions. The gene duplication analysis suggests that those two CaNFYB gene copies were evolutionary duplicated prior to the Solanaceae divergence. The presence of *P. patens* (moss) sequences are clearly distinct from the rest, underlining moss’s early divergence from the lineage leading to flowering plants (Figure 4).

The second clade (C2) is a large and diverse clade mainly characterized by genes from Arabidopsis (AT), potato (PGSC), tomato (Solyc), and pepper (XP) but not Physcomitrella (Pp), suggesting that the genes within these clades either emerged after the divergence of moss from the lineage leading to these vascular plants or not yet reported. The subclade C2.1 features the Arabidopsis gene AT5G47670.1 presenting NFYB subunit 6. It further diverges into a subclade that includes *CaNFYB10* and Solyc genes. This subclade then branches out to two deeper branching, one with genes from the PGSC and Solyc group clustered with *CaNFYB16*, and another predominantly presented by PGSC and Solyc genes only. The subclade C2.2 begins with a grouping of Arabidopsis gene AT1G21970.1 presenting NFYB subunit 9 that branches out with a large subclade. This extensive subclade contains PGSC and Solyc genes closely related to each other, paired with four duplicated CaNFYB genes (*12*, *13*, *14,* and *17*), the latter in which is not yet localized on a position in the pepper genome chromosomes (Figure 4).

The third clade (C3) is the largest and most diverse clade that mostly includes all the studied species with multiple sub-subclades that spotlight intricate associations across these plants. The subclade C3.1 includes two minor subclades, one feature the Arabidopsis gene AT2G13570.1 presenting NFYB subunit 7, this minor subclade consists of PGSC, Solyc, and *CaNFYB06* genes and it branches to another Arabidopsis gene AT2G47810.1 presenting NFYB subunit 5. Further branching within reveals tightly grouped PGSC and Solyc genes that leads to a complex subclade consisting of PGSC, Solyc, and XP gene copies (*CaNFYB09*). Further branching showed that the *CaNFYB18* and *CaNFYB19* with an estimated gene duplication event were grouped solely with PSGC gene. A deeper exploration showcases a dense interaction of genes from all four plants: Arabidopsis AT1G09030.1 presenting NFYB subunit 4 along with potato, tomato, and pepper gene copies (i.e., *CaNFYB01*). Remarkably, this minor subclade shows a notable mixture of genes from different species, indicating potential evolutionary conservation or shared evolutionary pressures among these plants for the NFYB transcription factor family (Figure 4).

The subclade, denoted as C3.2, primarily initiates with a branch distinct to the Solanaceae family, encompassing genes from PGSC, Solyc, and *CaNFYB04*. This subclade subsequently bifurcates into two intricate minor subclades. Both these divisions originate from a series of Pp NFYB genes, illustrating evident gene duplication events, and further diversify to include a comprehensive array of genes from Arabidopsis, PGSC, Solyc, and XP. The first of these minor subclades prominently features the Arabidopsis gene AT4G14540.1, representative of the NFYB subunit 3. This gene coexists in close evolutionary proximity with genes from PGSC, Solyc, and *CaNFYB02*. Additionally, another branch within this subclade highlights the Arabidopsis gene AT5G47640.1, which signifies the NFYB subunit 2, co-branching with genes from PGSC, Solyc, and *CaNFYB15*. Conversely, the subsequent minor subclade showcases a notable evolutionary trait with the Arabidopsis genes AT2G37060.1 and AT3G53340.1. These genes, indicative of duplication events, correspond to the NFYB subunits 8 and 10, respectively. Accompanying these genes in the same clade are the PGSC, Solyc, and the pepper genes *CaNFYB05* and *CaNFYB11* (Figure 4).

### 3.6 CaNFYB gene expression profiles in response to salinity stress

**FIGURE 5:**
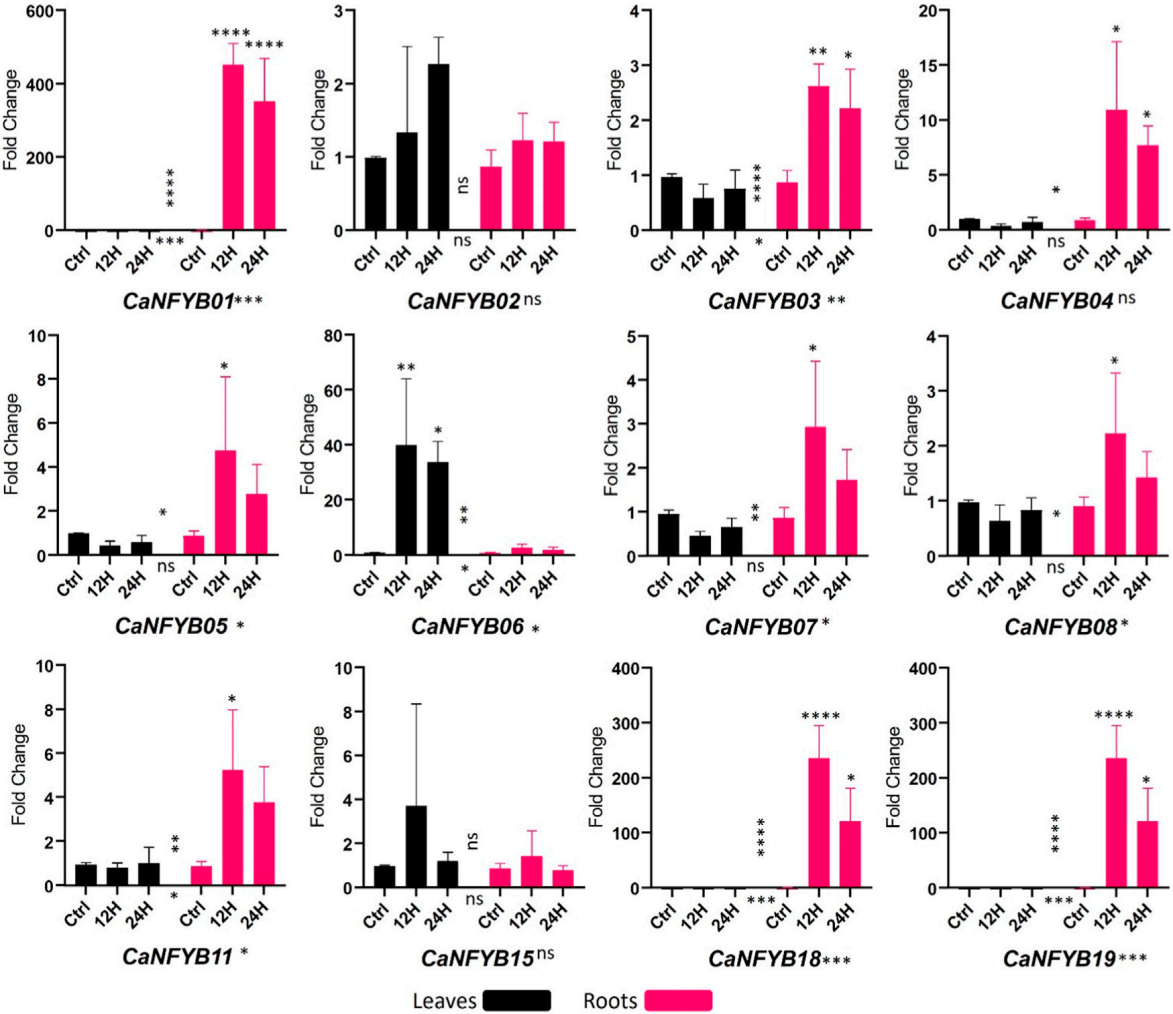
Expression profiles of CaNFYB genes across two time points (12 h and 24 h) in leaf and root tissues under salinity stress. Variations highlight time, tissue, and their interactions. ****: *p*-value< 0.0001, ***: *p*-value <0.001, **: *p*-value < 0.01; *: *p*-value < 0.05, NS: *p*-value > 0.05.

In response to salinity stress, the expression dynamics of the CaNFYB gene family were thoroughly investigated across two temporal intervals, 12 h and 24 h, in both leaf and root tissues of the plant. The significance of expression changes was meticulously assessed using a two-way ANOVA complemented by a Tukey multiple comparison *post hoc* test. The CaNFYB genes in pepper plants exhibit diverse transcriptional dynamics in response to salinity stress, which can be better elucidated when examining each factor—time, tissue, and their interaction (Figure 5).

With respect to the time dependent response, *CaNFYB01*, *CaNFYB03*, *CaNFYB06*, *CaNFYB11*, *CaNFYB18*, and *CaNFYB19* all displayed significant temporal variations in their expression profiles. Especially notable were *CaNFYB01*, *CaNFYB18*, and *CaNFYB19*, which displayed strong significance with *p*-values <0.001 or <0.0001, suggesting these genes respond promptly during the early phases of salinity stress. While *CaNFYB02*, *CaNFYB04*, *CaNFYB05*, *CaNFYB07*, *CaNFYB08*, and *CaNFYB15* either had no significant time-based changes or only moderate temporal dynamics, showing a stabilized or delayed responses to the stress over the time frames observed (Figure 5).

Based on the tissue type, pronounced responses were observed for *CaNFYB01*, *CaNFYB03*, *CaNFYB06*, *CaNFYB07*, *CaNFYB08*, *CaNFYB11*, *CaNFYB18*, and *CaNFYB19* in the roots. The heightened significance in the roots of *CaNFYB01*, *CaNFYB03*, *CaNFYB18*, and *CaNFYB19* (with *p*-values ranging from <0.01 to <0.0001) underscores these genes activity in root responses to salinity. In leaves, *CaNFYB06* stood out with substantial upregulation, indicating a specific function in foliar responses to salinity stress (Figure 5).

A complex interplay between temporal and spatial expression dynamics was noted for *CaNFYB01*, *CaNFYB03*, *CaNFYB06*, *CaNFYB07*, *CaNFYB08*, *CaNFYB11*, *CaNFYB18*, and *CaNFYB19*. Their significant interaction effects indicate that the gene expression responses to salinity in these members are not simply the sum of time and tissue effects but involve more intricate relationships. In contrast, *CaNFYB02*, *CaNFYB04*, *CaNFYB05*, and *CaNFYB15* either showed no significant interactions or displayed more subtle modulations, suggesting that their responses might be more direct and less influenced by the interplay of time and tissue. However, in the analyzed dataset, the transcriptional profiles of *CaNFYB09*, *CaNFYB10*, *CaNFYB12*, *CaNFYB13*, *CaNFYB14*, *CaNFYB16*, and *CaNFYB17* exhibited an absence of detectable RNA expression levels under the experimental conditions. And both *CaNFYB02* and *CaNFYB15* manifested no statistically significant modulation in their transcriptional activities in response to the evaluated salinity stress conditions under any condition (Figure 5).

## 4 Discussion

The *in silico* identification of 19 CaNFYB genes in the pepper genome provides a comprehensive overview of this gene family in *C. annuum*. The wide range of molecular weight values observed among these proteins is reminiscent of the variability seen in other plant species, such as Arabidopsis and rice, highlighting the diversity and potential functional versatility of this transcription factor family (Petroni et al., 2012). In contrast to fungi and animals, plants typically possess an average of ∼10 NF-YB genes (Nelson et al., 2007). The number of subunits identified in pepper aligns with the numerous copies of the NFYB family found in other higher plants, despite the discrepancies observed in CaNFYB copies among published pepper genomes. For instance, within the Solanaceae family, potato was reported to have 22 NFYB genes (Li et al., 2021), while tomato had 29 (Li et al., 2016). However, in lower plants, such as *P. patens*, only 9 copies were reported (Zhang et al., 2015). These variations in gene copy numbers across plant species reflect a consistent expansion pattern likely linked to the evolution and diversification of land plants (Laloum et al., 2013).

All copies were retained under the condition of annotating the NFYB domain and confirmed with the secondary structure analysis. Notably, NFYB proteins exhibit a distinctive structure within the heterodimeric NFY complex. Unlike NFYA, NFYB contains a histone fold domain (Petroni et al., 2012), and it possesses a DNA binding site along with two protein-protein interaction domains (Kahle et al., 2005) that bind to NFYA and NFYC proteins (Zhang et al., 2015). The CaNFYB proteins reveal an intricate balance between *α*-helices and *β*-sheets. This balance is paramount, as the secondary structure elements are pivotal for protein functionality, especially in transcription factors requiring specific conformations to interact with DNA (Riechmann et al., 2000). The observed similarities and variations in these structures among CaNFYB proteins may hint at both conserved and unique DNA binding domains or interaction surfaces, much like those reported in NFYB proteins of Arabidopsis (Gusmaroli et al., 2002), tomato (Li et al., 2016) and potato (Li et al., 2021).

Exploring the chromosomal locations and structural intricacies of the CaNFYB genes unveils significant insights into their evolution. Their varied distribution across the pepper chromosomes resembles patterns observed in other genomes, hinting at complex evolutionary events, including gene duplications and translocations (Cannon et al., 2004). Notably, CaNFYB copies are situated on seven out of the 12 pepper chromosomes, a distribution more constrained than that observed in tomato (which spans 11 out of 12 chromosomes; Li et al., 2016) and potato (across all 12 chromosomes; Li et al., 2021). The presence of gene clusters may suggest tandem duplication events, a well-recognized driver of gene family expansion. In our study, we identified a tandem duplication on chromosome 7 in pepper. In contrast, tomato exhibited a tandem duplication on chromosome 5 (Li et al., 2016), while potato displayed them on chromosomes 3 and 5 (Li et al., 2021). These differences in the location of tandem duplications highlight the unique evolutionary paths and dynamics of the CaNFYB gene family in pepper compared to other Solanaceae species.

The observed variation in exon-intron structures among CaNFYB genes aligns with findings in other species (Zhang et al., 2015; Li et al., 2016; Li et al., 2016) and could be indicative of evolutionary diversification, where conserved structures denote ancestral functions, while unique structures hint at acquired roles (Wang et al., 2017). The evolutionary analysis of the CaNFYB genes traces the intricate relationships and potential ancestral connections among several plant species. The observed bifurcations and clades echo the evolutionary patterns of the NFYB gene family reported in diverse plant lineages (Siefers et al., 2009) and were found homogenized with the clusters reported by Li et al. (2016) and Li et al. (2021). The prominent ancestral clades and the moss sequence’s distinct positioning reaffirm the evolutionary divergence between non-vascular and vascular plants. The inclusion of genes from other Solanaceae members provides a comparative framework to hypothesize about gene emergence, diversification, and potential functional conservation (Nelson and Werck-Reichhart, 2011).

Potential cis-element analysis in the promoter regions of CaNFYB genes offers a window into the putative regulatory mechanisms at play. The prevalent light-responsive elements found mirror findings in other plants, highlighting the significant role light plays in regulating plant gene expression (Hudson and Quail, 2003). The discovery of multiple abscisic acid and MeJA responsive elements resonates with the established importance of these hormones in plant stress responses (Cutler et al., 2010; Wasternack and Hause, 2013). In Arabidopsis, *AtNFYB*2 has been found to be upregulated in response to osmotic stress (Chen et al., 2007). In barley (*Hordeum vulgare*), the transcript levels of *HvNFYB*3 were induced by drought stress, while *HvNFYB*1 and *HvNFYB*4 exhibited a significant decrease in transcription levels following the application of abscisic acid (ABA). Additionally, *HvNFYB*5 displayed a notable increase in transcription levels in response to high salt stress (Liang et al., 2012). The observed variability in TF binding sites among the CaNFYB genes suggests diverse roles and interactions in various biological processes including the tolerance to abiotic stresses (e.g., Salinity and drought). In particular, the diversified TF binding sites in *CaNFYB18* and *CaNFYB19* suggest an involvement in multiple signaling pathways. Such multifunctional genes, regulated by diverse cis-elements, are often key nodes in regulatory networks, ensuring the plant’s adaptability to varied environmental conditions (Ganie et al., 2017).

Delving into the expression dynamics of CaNFYB genes under salinity stress, the observed variability resonates with the recognized modulatory roles of NFYBs in abiotic stress responses (Leyva-González et al., 2012). Time-dependent responses of specific genes, such as *CaNFYB01*, *CaNFYB18,* and *CaNFYB19*, mirror patterns seen in other plants where early-response genes act as primary regulators in stress signaling pathways (Shinozaki and Shinozaki, 2006). The pronounced tissue-specific responses, especially in roots, align with the established understanding that roots serve as primary sensors during salinity stress, regulating both root and shoot adaptive responses (Munns and Tester, 2008). The absence of detectable expression in certain genes like *CaNFYB09* and *CaNFYB10* hints at the potential of these genes being triggered under specific untested conditions or specific developmental stages, a phenomenon observed in several plant transcription factor families (Riechmann et al., 2000; Rushton et al., 2008; Wang et al., 2020; Dai et al., 2021; Wang et al., 2022).

In tomato, an in-depth investigation has revealed the significant involvement of several NFY genes, particularly those belonging to the NFYB and NFYA subgroups, in the process of fruit ripening, as documented by Li et al. (2016). They conducted a comprehensive quantification analysis of four tomato NFYB genes, revealing their upregulation in both leaves and roots during tomato plant development. Notably, *SlNFYB3* exhibited expression in both tissues, with higher levels in roots. This gene is a homolog of *CaNFYB15*, which, interestingly, displayed stable expression in both tissues with no significant changes under salinity treatment. This suggests a pivotal role for *CaNFYB15* in plant development rather than in conferring abiotic stress tolerance. Similarly, in potato, Li et al. (2021) quantified the expression of *StNFYB14*, a homolog of the same gene, under salinity conditions. They found that *StNFYB14* was upregulated at 1, 6, and 12 h of salinity exposure. Another gene, *StNFYB22*, which is a homolog of *CaNFYB08*, exhibited the highest expression level under salinity stress, but notably, this upregulation was most pronounced after 1 h of exposure (approximately 7-fold higher than the control), decreasing to 3-fold higher after 12 h of exposure. In contrast, in pepper, the *CaNFYB*08 genes displayed stable expression in both tissues, with no significant changes observed under salinity treatment. These findings suggest that different regulatory mechanisms may govern the expression and regulation of NFYB genes among different genera within the Solanaceae family. These observations highlight the intricate and context-dependent nature of NFYB gene regulation in response to environmental stimuli and suggest that the roles of these genes can vary significantly among species, even within the same plant family.

Notably, in the moss species *P. patens*, specific members of the PpNFY family exhibited diverse expression patterns in response to abiotic stress. For instance, the *PpNFYB*6 gene displayed a significant peak in expression after 24 h of Mannitol treatment. In contrast, the *PpNFYB1* gene exhibited upregulation after short durations of drought stress (2 h) or ABA treatments (12 h), suggesting its potential involvement in early stress responses. Intriguingly, when examining an induced *P. patens* mutant, ab3i (associated with the ABI3-Dependent Pathway and responsive to ABA), the *PpNFYB1* gene showed significantly higher expression in the mutant compared to the wild type. This finding parallels the observation that its homolog, *CaNFY03*, displayed significant expression in pepper roots as opposed to leaves during 12 h and 24 h of salinity treatment. In contrast, the *PpNFYB3* and *PpNFYB8* genes in *P. patens* appeared to function as negative regulators, as their expression decreased under salinity stress, as reported by Zhang et al. (2015). This stands in contrast to their homologs in pepper, *CaNFYB07*, and *CaNFYB08*, which exhibited complex interactions between time and tissue, with slightly higher expression levels in roots than in leaves. It is important to note that these differences may be attributed to the moss’s unique nature, as mosses lack the typical root structure found in higher plants. These observations emphasize the dynamic and context-dependent nature of NFYB gene regulation in response to abiotic stress and highlight potential variations in regulatory mechanisms between mosses and higher plants like pepper.

Further expanding out of the Solanaceae family, a study on *Eucalyptus grandis* (Family Myrtaceae) discovered 23 EgNFYB genes, segregating them into LEC-1 and non-LEC1 types based on phylogenetic analysis. Among these, *EgNFYB4/6/13/19/23* were found to be particularly active in responses to salinity, heat, and cold stresses, highlighting their essential roles in abiotic stress responses in eucalyptus (Dai et al., 2021).

Moreover, the NFY transcription factors, especially the NFYB subfamily, have been central to multiple physiological and developmental processes in plants. In Arabidopsis, *AtNFYB2*, and *AtNFYB3* are associated with the regulation of flowering time and *AtNFYB1* is associated with the enhancement of drought resistance (Nelson et al., 2007; Kumimoto et al., 2010) In maize, *ZmNFYB2* plays a pivotal role in the photoperiod-dependent initiation of flowering (Kumimoto et al., 2010). Overexpression of *ZmNFYB2* not only improved the drought resistance but also increased production in withholding water conditions (Nelson et al., 2007). In legumes, *Medicago truncatula* NFYB genes, specifically in association with *MtNFYA1* and *MtNFYA2*, are integral for the symbiotic interactions with *Sinorhizobium meliloti* (Baudin et al., 2015). A similar interaction pattern was observed in the common bean (*Phaseolus vulgaris*), emphasizing the evolutionary conservation of these NFY protein complexes in leguminous plants.

## 5 Conclusion

The study of the CaNFYB gene family in *C. annuum* unveils their integral roles in vital plant physiological responses, especially during environmental stress. Particularly, genes such as *CaNFYB01*, *CaNFYB18*, and *CaNFYB19* display profound transcriptional dynamics during the initial phases of salinity stress, indicating their central part in initiating stress defense mechanisms. Moreover, a distinct tissue-specific response was evident, exemplified by the marked upregulation of *CaNFYB06* in leaves, suggesting an adaptive mechanism tailored to specific organ needs. An evolutionary deep dive underscored the ancestral functions and divergence pathways of the CaNFYB genes, with genes like *CaNFYB07* and *CaNFYB08* possibly maintaining pivotal ancestral roles within the Solanaceae lineage. The diverse transcription factor binding sites, especially predominant in genes like *CaNFYB18* and *CaNFYB19*, hint at their multifaceted roles in different signaling pathways, a mechanism likely to boost their adaptability under varied conditions. Interestingly, several CaNFYB genes did not register detectable expression under the conditions tested, leading to speculation about their activation under specific untested circumstances or during certain developmental phases. Collectively, the insights have drawn spotlight on the multifunctional roles and evolutionary significance of the CaNFYB gene family in pepper plants.

## Data Availability

The original contributions presented in the study are included in the article/Supplementary Material, further inquiries can be directed to the corresponding author.
