# Supplementary material for: Evolutionary insights and expression dynamics of the CaNFYB transcription factor gene family in pepper (Capsicum annuum) under salinity stress
Source: Front Genet. 2023 Nov 2;14:1288453. doi: 10.3389/fgene.2023.1288453 (PMC10652888; doi:10.3389/fgene.2023.1288453)
Supplement: Supplementary file 1 [file Table1.docx]

Evolutionary Insights and Expression Dynamics of the *CaNFYB* Transcription Factor Gene Family in Pepper (*Capsicum annuum*) under Salinity Stress

**Suppl. Table 1. Primers for Amplification of CDS from *CaNFYB* Gene Variants.** The table provides information on the CDS IDs (pertaining to specific protein sequences of *CaNFYB* gene variants) and their corresponding primer sequences. Detailed data include the primer ID, sequence, expected amplicon size (in base pairs), and melting temperature (Tm) of the primer.

| CDS ID | Primer ID | 5’-Sequence-3’ | Expected Size (bp) | Tm (°C) |
| --- | --- | --- | --- | --- |
| CaNFYB05 - XP_016550674.1 | 05F | GATGGAGGGAGATGCCAAGG | 95 | 60 |
| CaNFYB05 - XP_016550674.1 | 05R | TGAACTGCGTATTGGACCCA |  | 59 |
| CaNFYB18 - XP_047269333.1 | 18F | TTCTTCCACCAAATGCCAAGA | 97 | 58 |
| CaNFYB18 - XP_047269333.1 | 18R | TCACATGCTTCACCTGTCACA |  | 60 |
| CaNFYB19 - XP_047269351.1 | 19F | TTCTTCCACCAAATGCCAAGA | 97 | 58 |
| CaNFYB19 - XP_047269351.1 | 19R | TCACATGCTTCACCTGTCACA |  | 60 |
| CaNFYB17 - XP_047260967.1 | 17R | TCTTCATCATTCTCCGCCACT | 100 | 59 |
| CaNFYB17 - XP_047260967.1 | 17F | GGTACCCGCATTTTTCACCA |  | 59 |
| CaNFYB02 - XP_016541295.1 | 02F | TGGGGAGAAGTGGGGAGAAA | 101 | 60 |
| CaNFYB02 - XP_016541295.1 | 02R | CATACCGCCAGTACCACCAC |  | 61 |
| CaNFYB13 - XP_016579564.2 | 13R | TCGCCACTATGTTTTCCTTCCT | 120 | 60 |
| CaNFYB13 - XP_016579564.2 | 13F | TGCCCATCAACAGTGTACCA |  | 59 |
| CaNFYB10 - XP_016572034.1 | 10F | AGGTGGTGGAGGCTTTCATG | 125 | 60 |
| CaNFYB10 - XP_016572034.1 | 10R | TGTTGTGGTTTGTTGGGGGA |  | 60 |
| CaNFYB07 - XP_016555943.1 | 07F | GAGCCGGGAAGTGCAGTAAT | 133 | 60 |
| CaNFYB07 - XP_016555943.1 | 07R | TCTGGATCTTGCTGCTTGGG |  | 60 |
| CaNFYB16 - XP_047256461.1 | 16F | GCAGAAGATGTGCTTTGGGC | 152 | 60 |
| CaNFYB16 - XP_047256461.1 | 16R | AACACTCCGCGTCTCAACAT |  | 60 |
| CaNFYB12 - XP_016579563.2 | 12F | GCCTGGTATTGTTCCACCCA | 172 | 60 |
| CaNFYB12 - XP_016579563.2 | 12R | TTCATCATTCTCCGCCACCA |  | 59 |
| CaNFYB03 - XP_016542646.2 | 03F | AGTCATGAGAGTGGTGGGGA | 190 | 60 |
| CaNFYB03 - XP_016542646.2 | 03R | TTGCCTCGCTGGTGATGAAA |  | 60 |
| CaNFYB11 - XP_016572314.1 | 11F | CCATGAGAGTGGTGGTGGTG | 194 | 60 |
| CaNFYB11 - XP_016572314.1 | 11R | TTGCCTCGCTGGTGATGAAA |  | 60 |
| CaNFYB04 - XP_016549735.1 | 04F | AATAGCCCGGTTGGAAGTCC | 196 | 60 |
| CaNFYB04 - XP_016549735.1 | 04R | CTTCGCCCGTGATGAAGCTA |  | 60 |
| CaNFYB01 - XP_016538616.1 | 01F | CTTGCCACCAACAGCCAAAA | 197 | 60 |
| CaNFYB01 - XP_016538616.1 | 01R | ACCTCAACATGACCTCTGCA |  | 59 |
| CaNFYB09 - XP_016567970.2 | 09F | ACTCAAGCGAAGAAGGAGGG | 209 | 59 |
| CaNFYB09 - XP_016567970.2 | 09R | GTTGACCGTCTTGCGCTTTT |  | 60 |
| CaNFYB14 - XP_016579565.2 | 14F | TGTCGACACAGTAAAGGCGA | 223 | 59 |
| CaNFYB14 - XP_016579565.2 | 14R | AGCAGGCCTCAAATAGTGCA |  | 60 |
| CaNFYB08 - XP_016563434.1 | 08F | GTGTTGCCCGAGATACCCAA | 239 | 60 |
| CaNFYB08 - XP_016563434.1 | 08R | ATTGCTCCACTTCCCAGCTC |  | 60 |
| CaNFYB06 - XP_016552944.2 | 06R | ACGCATTCTTGAACCGTCTCT | 244 | 60 |
| CaNFYB06 - XP_016552944.2 | 06F | ATGGACGAAATGTGGCTGCT |  | 60 |
| CaNFYB15 - XP_016581428.1 | 15F | TGGGCAATGACAACTCTTGGT | 245 | 60 |
| CaNFYB15 - XP_016581428.1 | 15R | ACTCCTGCACCATTCTCAGC |  | 60 |

**Suppl. Table 2.** **Genomic Details and Annotations for *CaNFYB* Gene Family in *Capsicum annuum* (Bell Pepper) across different genomic assemblies.** The table encapsulates comprehensive genomic details pertaining to the *CaNFYB* gene family in Capsicum annuum. Information includes gene and protein IDs, chromosomal location, orientation, associated subunit, and quality annotations from various databases. This compilation aids in the in-depth understanding and comparison of *CaNFYB* genes within the bell pepper genome.

| ID | Reference | NCBI accession | GeneID | ProteinID | TranscriptID | Min | Max | Chr | Sense | SubunitID | GeneIDV2 | GradeV2 | EnsID | GradeEns | PlantTFdbID | GradeTFdb |
| --- | --- | --- | --- | --- | --- | --- | --- | --- | --- | --- | --- | --- | --- | --- | --- | --- |
| CaNFYB01 | UCD10Xv1.1 | NW_025847490.1 | LOC107839585 | XP_016538616.1 | XM_016683130.2 | 31833 | 32536 | ctg4021 | - | 4 | Capana08g001486 | 1 | PHT63196 | 1 | CA08g12240 | 1 |
| CaNFYB02 | UCD10Xv1.1 | NC_061119.1 | LOC107841985 | XP_016541295.1 | XM_016685809.2 | 203579279 | 2E+08 | 9 | + | 3 | Capana09g000479 | 1 | PHT73254 | 1 | CA09g14120 | 1 |
| CaNFYB03 | UCD10Xv1.1 | NC_061119.1 | LOC107843031 | XP_016542644.2 | XM_016687160.2 | 11486454 | 1.1E+07 | 9 | + | 10 | Capana09g002063 | 0.964 | PHT71968 | 0.994 | CA09g03270 | 0.84 |
| CaNFYB04 | UCD10Xv1.1 | NC_061122.1 | LOC107849692 | XP_016549735.1 | XM_016694249.2 | 55988382 | 5.6E+07 | 12 | - | 3 | Capana12g001765 | 1 | PHT65720 | 1 | CA12g12150 | 1 |
| CaNFYB05 | UCD10Xv1.1 | NC_061122.1 | LOC107850574 | XP_016550674.1 | XM_016695188.2 | 140223010 | 1.4E+08 | 12 | + | 10 | Capana12g001654 | 0.994 | PHT65745** | 0.853 | CA12g10990 | 0.853 |
| CaNFYB06 | UCD10Xv1.1 | NC_061121.1 | LOC107852425 | XP_016552944.2 | XM_016697458.2 | 174480201 | 1.7E+08 | 11 | - | 3-like | Capana00g001840 | 0.991 | PHT68352 | 0.843 | CA11g10090 | 0.993 |
| CaNFYB07 | UCD10Xv1.1 | NC_061116.1 | LOC107855438 | XP_016555943.1 | XM_016700457.2 | 208813893 | 2.1E+08 | 6 | + | Dr1 homolog | Capana00g004693 | 0.937 | PHT79567 | 1 | CA06g18120 | 1 |
| CaNFYB08 | UCD10Xv1.1 | NC_061113.1 | LOC107862382 | XP_016563434.1 | XM_016707948.2 | 250696219 | 2.5E+08 | 3 | + | Dr1 homolog | Capana03g000957 | 1 | PHT88188 | 1 | CA03g28530 | 1 |
| CaNFYB09 | UCD10Xv1.1 | NC_061113.1 | LOC107866402 | XP_016567970.2 | XM_016712484.2 | 32218308 | 3.2E+07 | 3 | + | 5 | Capana03g003587 | 0.997 | PHT86228 | 1 | CA03g08630 | 1 |
| CaNFYB10 | UCD10Xv1.1 | NC_061115.1 | LOC107870124 | XP_016572034.1 | XM_016716548.2 | 1983102 | 1987384 | 5 | - | 6 | NA | NA | PHT62530 | 0.908 | CA05g01770 | 0.824 |
| CaNFYB11 | UCD10Xv1.1 | NC_061115.1 | LOC107870337 | XP_016572314.1 | XM_016716828.2 | 10618905 | 1.1E+07 | 5 | + | 1 | Capana05g000545 | 0.991 | PHT80962 | 0.994 | CA05g04100/CA04g06370** | 93.4%/42.2% |
| CaNFYB12 | UCD10Xv1.1 | NC_061117.1 | LOC107877427 | XP_016579563.2 | XM_016724077.2 | 225133015 | 2.3E+08 | 7 | - | 6-like | Capana07g002379 | 0.923 | PHT77397 | 1 | NA | NA |
| CaNFYB13 | UCD10Xv1.1 | NC_061117.1 | LOC107877428 | XP_016579564.2 | XM_016724078.2 | 225138757 | 2.3E+08 | 7 | - | 9 | Capana07g002380 | 0.863 | PHT77398 | 0.889 | NA | NA |
| CaNFYB14 | UCD10Xv1.1 | NC_061117.1 | LOC107877429 | XP_016579565.2 | XM_016724079.2 | 225141938 | 2.3E+08 | 7 | - | 6 | Capana07g002381 | 0.919 | PHT77399 | 0.923 | NA | NA |
| CaNFYB15 | UCD10Xv1.1 | NC_061117.1 | LOC107878806 | XP_016581426.1 | XM_016725943.2 | 225085346 | 2.3E+08 | 7 | - | 3 | Capana07g002368 | 1 | PHT77378 | 1 | CA07g20350 | 1 |
| CaNFYB16 | UCD10Xv1.1 | NC_061122.1 | LOC107850352 | XP_047256461.1 | XM_047400505.1 | 1137336 | 1134587 | 12 | + | 6 | Capana12g002889 | 0.895 | PHT64593 | 0.959 | CA12g00340 | 0.963 |
| CaNFYB17 | UCD10Xv1.1 | NW_025882730.1 | LOC124894287 | XP_047260967.1 | XM_047405011.1 | 1493 | 2575 | ctg72777 | - | 9-like | Capana07g002376 | 0.927 | PHT77395 | 1 | NA | NA |
| CaNFYB18 | UCD10Xv1.1 | NC_061116.1 | LOC124899203 | XP_047269333.1 | XM_047413377.1 | 771819 | 772458 | 6 | - | 5-like | NA | NA | PHT63251 | 0.802 | CA05g15010 | 1 |
| CaNFYB19 | UCD10Xv1.1 | NC_061116.1 | LOC124899207 | XP_047269351.1 | XM_047413395.1 | 1207873 | 1207234 | 6 | + | 5-like | NA | NA | PHT63251* | 0.802 | CA05g15010* | 1 |

* no copy matches but match to a repeated copy is annotated in the same reference genome; ** two copies matches the one gene.
